# Suicidal thoughts and behaviors (STB) among psychiatric emergency patients at the emergency unit of a university hospital in Belgium (UZ Leuven). A twenty year perspective using cross-sectional data.

**DOI:** 10.1192/j.eurpsy.2024.304

**Published:** 2024-08-27

**Authors:** L. Van Eldere, S. Claes, W. Voorspoels, M. Sabbe, R. Bruffaerts

**Affiliations:** KU Leuven, Leuven, Belgium

## Abstract

**Introduction:**

Suicidal thoughts and behaviors (STB) are a serious public health problem. Suicide prevention programs have been established over the years, but many people who are suicidal do not seek treatment, and when they do, they end up in low-threshold sectors such as the Emergency Department in general hospitals. Previous studies about STB at the ED are mostly narrative, rather than a date-driven approach and limited in sample size .

**Objectives:**

This study describes the prevalence and evolution over time of suicidal ideation (SI) and suicidal attempts (SA) in terms of sociodemographic, clinical and service use variables of the psychiatric patient referred to the Emergency Department of the University Hospital Gasthuisberg (Leuven, Belgium) over a 20 year period.

**Methods:**

During a 20 year period (2002-2022), all patients with a psychiatric referral to the Psychiatric Emergency Department (PED) of the University Hospital Gasthuisberg (Leuven) were included (N˜18.000). We use descriptive statistics to summarize the data set, focusing on STB in terms of sociodemographic, clinical and service use variables.

**Results:**

Around 1/10 patients presents with SA; another 1/5 with SI. Despite several reforms, SI and SA have remained relatively stable over the years. Notably, there is a higher prevalence of referrals for females in both SI and SA compared to males. However, there has been a notable increase in male SA cases over time. In the age group 36-49, both sexes exhibit the highest percentages of SI and SA cases, with exception for women in SI, where the age category 18-25 has the most referrals. Approximately one-third of male patients referred with STB have never accessed outpatient care, underscoring a critical gap in mental health services for this demographic.

**Conclusions:**

Despite several reforms in mental health care, the PED remains a major entry point into mental healthcare for large proportions of STB patients.

**Disclosure of Interest:**

None Declared

